# Supplementary material for: Reinforcement Learning Recruits Somata and Apical Dendrites across Layers of Primary Sensory Cortex
Source: Cell Rep. Author manuscript; Available in PMC 2020 Feb 5. (PMC7001879; doi:10.1016/j.celrep.2019.01.093)
Supplement: 1 [file NIHMS1548831-supplement-1.pdf]

Cell Reports, Volume *26*

Supplemental Information

Reinforcement Learning Recruits Somata and Apical Dendrites across Layers of Primary Sensory Cortex

Clay O. Lacefield, Eftychios A. Pnevmatikakis, Liam Paninski, and Randy M. Bruno

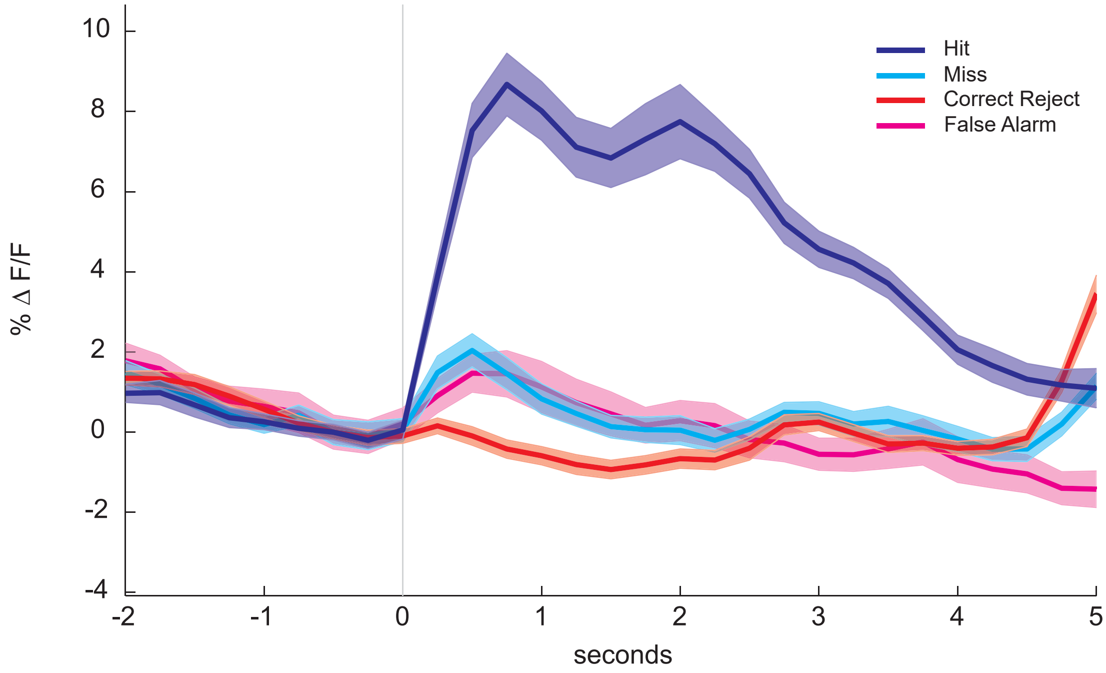

Figure S1, related to Figure 1: Trial averaged whole-frame calcium responses from all four trial outcomes (same data as Fig. 1F).

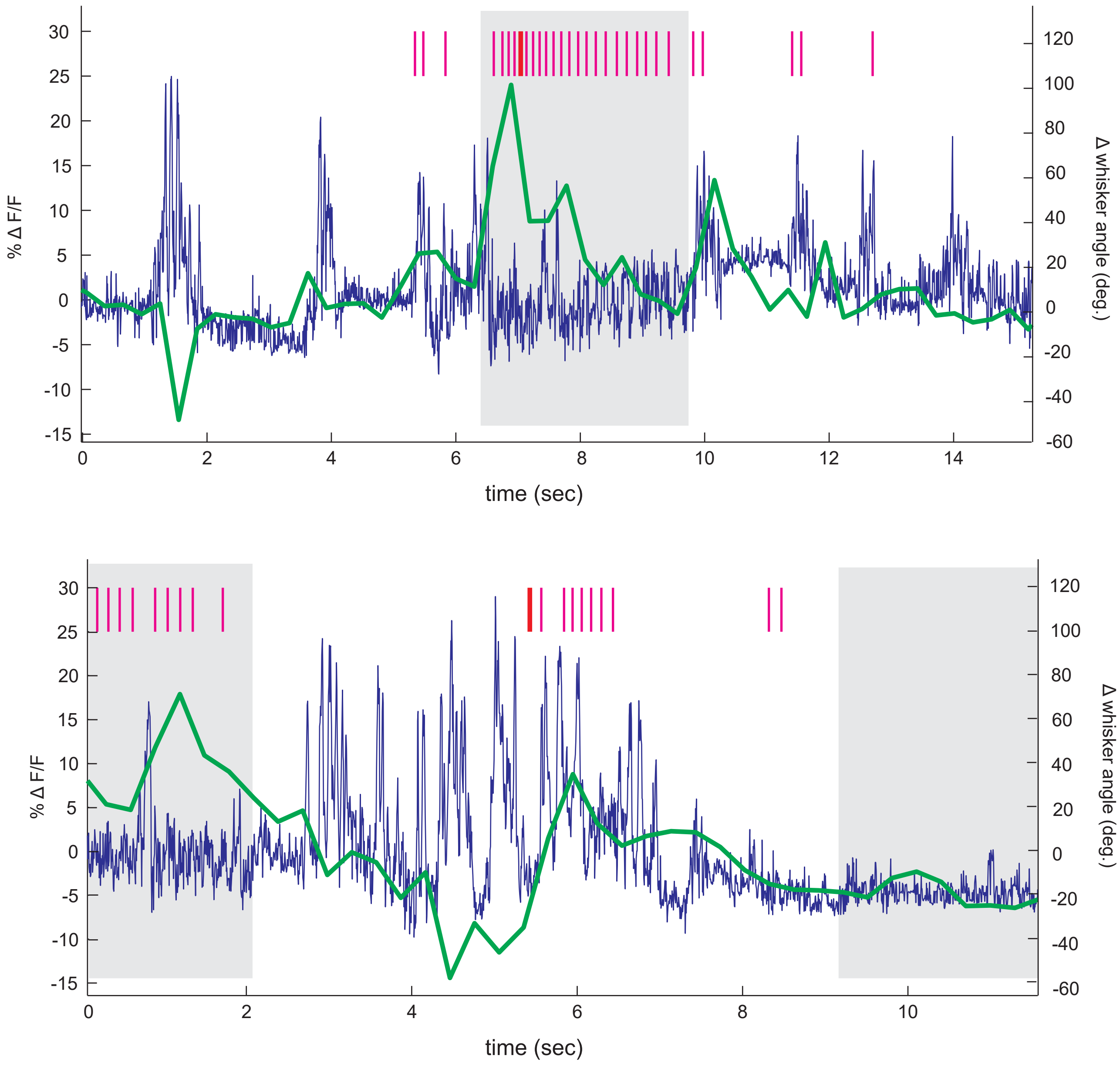

Figure S2, related to Figure 2: Whisker angle (blue) and Layer 5 dendritic calcium (green) during a single rewarded Go trial (top) and random ITI reward (bottom). Gray areas represent trial epochs, while red ticks indicate reward times and magenta ticks indicate lick times. Note that in bottom panel, spontaneous whisking during ITI is accompanied by decreases in calcium levels until the onset of the random ITI reward, which is accompanied by an increase in calcium influx.

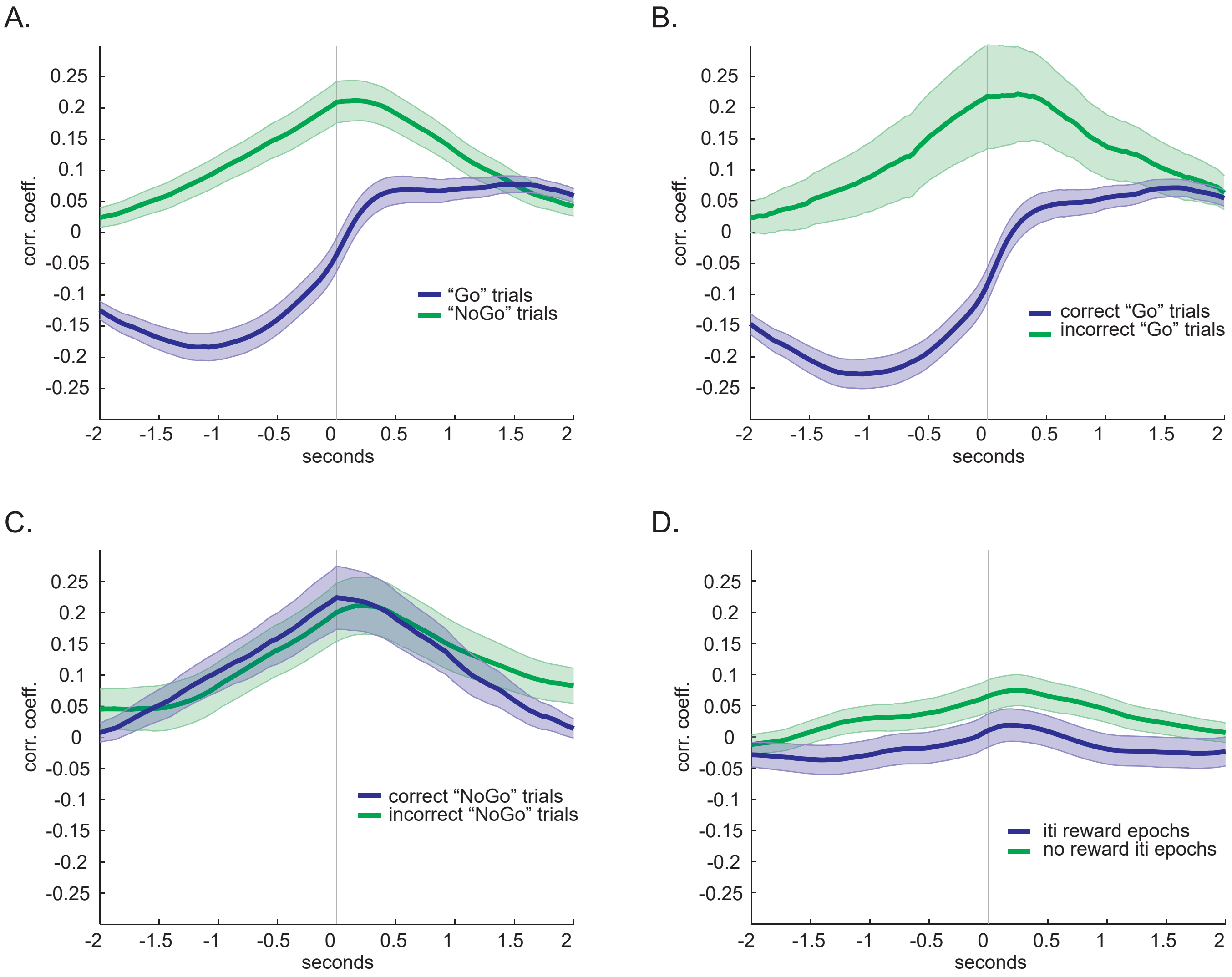

Figure S3, related to Figure 2: Whisking-calcium correlations during behavioral epochs.
(A) Correlation between whisker angle and whole-frame calcium during stimulus "go" trials (blue, n = 204 trials), compared with nogo trials (green, n = 157 trials).
(B) correct/hit (blue, n = 153 trials) vs. incorrect/miss (green, n = 35 trials) "go" trials
(C) correct reject (blue, n = 93 trials) vs. incorrect/false alarm (green, n = 63 trials) "nogo" trials
(D) ITI reward epochs (blue, n = 132 epochs) vs. normal ITI epochs with no random reward (green, n = 229 epochs). In all of these cases, trials with rewards show a decrease in overall correlation between whisking and calcium (n = 6 sessions in 2 mice).

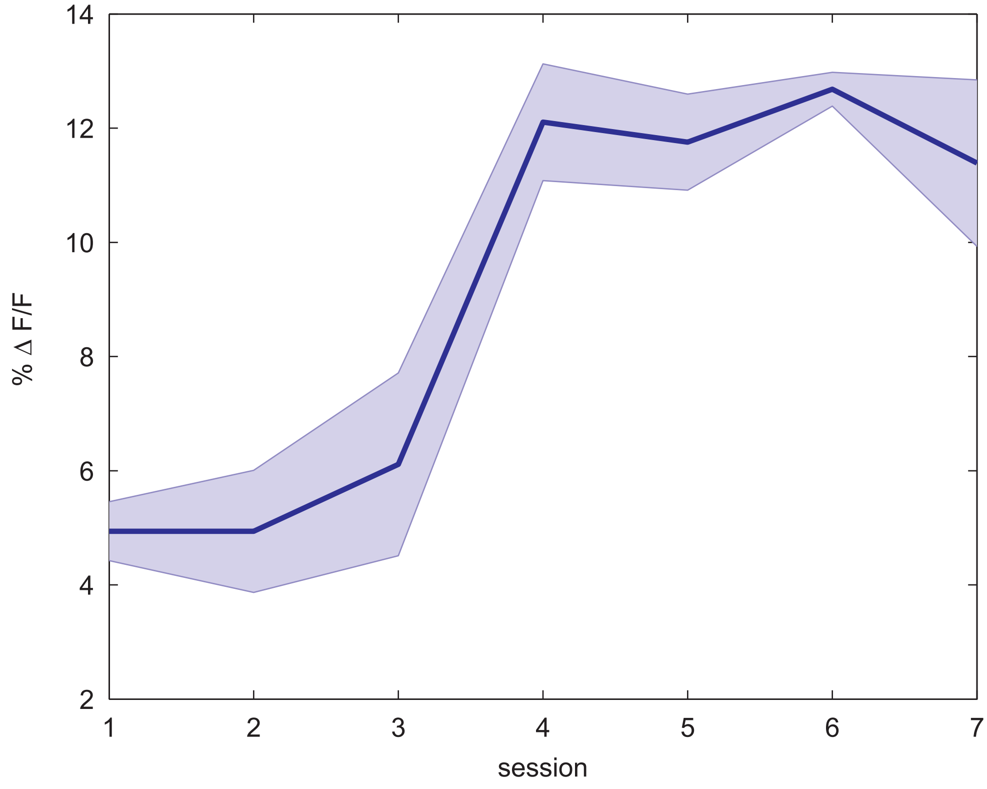

Figure S4, related to Figure 2: Average amplitude of the long-latency peak during trials over sessions (n = 3 animals). Error bound, SEM.

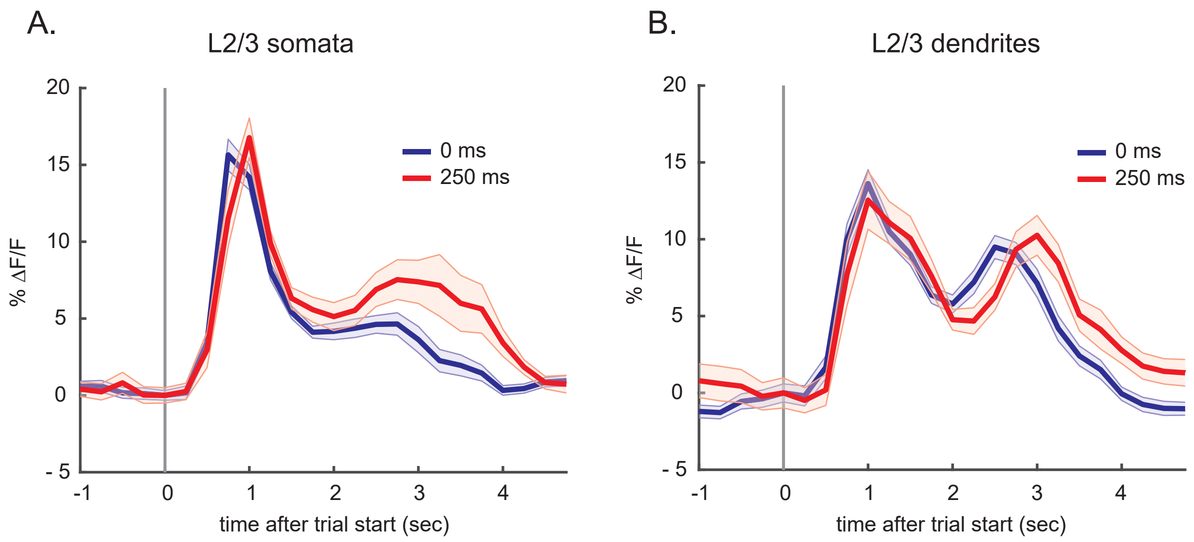

Figure S5, related to Figure 4: Reward tracking in L2/3 somata and dendrites.
(A) Whole-frame calcium signals in L2/3 somata to pole/"Go" trial rewards delayed by 0ms (blue) or 250ms (red).
(B) Whole-frame calcium signals in L2/3 dendrites to pole/"Go" trial rewards delayed by 0ms (blue) or 250ms (red).
