# Supplementary material for: Reinforcement Learning Recruits Somata and Apical Dendrites across Layers of Primary Sensory Cortex
Source: Cell Rep. Author manuscript; Available in PMC 2020 Feb 5. (PMC7001879; doi:10.1016/j.celrep.2019.01.093)
Supplement: 2 [file NIHMS1548831-supplement-2.pdf]

Cell Reports

Report

Reinforcement Learning Recruits Somata and Apical Dendrites across Layers of Primary Sensory Cortex

Graphical Abstract

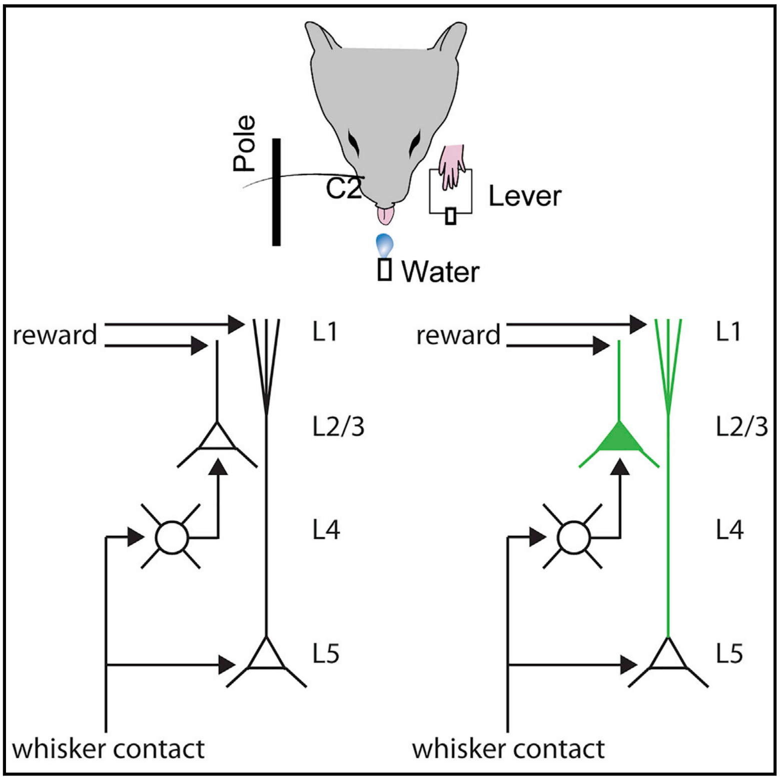

Highlights

- Events outside a cortical area's modality can trigger tuft-wide dendritic spikes
- Rewards modulate dendritic and somatic activity in layers 1, 2/3, and 5, but not 4
- Reward-related activity emerges gradually with training and is task specific
- Both expected and unexpected rewards can modulate primary sensory cortex

Authors

Clay O. Lacefield, Eftychios A. Pnevmatikakis, Liam Paninski, Randy M. Bruno

Correspondence

randybruno@columbia.edu

In Brief

Previously, the only known triggers of apical dendritic spikes were “bottom-up” events, such as appropriate sensory stimuli or an animal's location in space. Lacefield et al. show that reinforced associations are powerful triggers of apical dendrite activity and that reward can manipulate perceptions at their earliest stages of cortical processing.

Lacefield et al., 2019, Cell Reports *26*, 2000–2008
February 19, 2019 © 2019 The Author(s).
https://doi.org/10.1016/j.celrep.2019.01.093

OPEN ACCESS
CellPress

Cell Reports
Report

Reinforcement Learning Recruits Somata and Apical Dendrites across Layers of Primary Sensory Cortex

Clay O. Lacefield,[1,2] Eftychios A. Pnevmatikakis,[3,5] Liam Paninski,[1,2,3,4] and Randy M. Bruno[1,2,6,*]
[1]Department of Neuroscience, Mortimer Zuckerman Mind Brain Behavior Institute, Columbia University, New York, NY 10027, USA
[2]Kavli Institute for Brain Science, Columbia University, New York, NY 10027, USA
[3]Department of Statistics, Columbia University, New York, NY 10027, USA
[4]Grossman Center for the Statistics of Mind, Columbia University, New York, NY 10027, USA
[5]Present address: Center for Computational Mathematics, Flatiron Institute, New York, NY 10010, USA
[6]Lead Contact
*Correspondence: randybruno@columbia.edu
https://doi.org/10.1016/j.celrep.2019.01.093

SUMMARY

The mammalian brain can form associations between behaviorally relevant stimuli in an animal's environment. While such learning is thought to primarily involve high-order association cortex, even primary sensory areas receive long-range connections carrying information that could contribute to high-level representations. Here, we imaged layer 1 apical dendrites in the barrel cortex of mice performing a whisker-based operant behavior. In addition to sensory-motor events, calcium signals in apical dendrites of layers 2/3 and 5 neurons and in layer 2/3 somata track the delivery of rewards, both choice related and randomly administered. Reward-related tuft-wide dendritic spikes emerge gradually with training and are task specific. Learning recruits cells whose intrinsic activity coincides with the time of reinforcement. Layer 4 largely lacked reward-related signals, suggesting a source other than the primary thalamus. Our results demonstrate that a sensory cortex can acquire a set of associations outside its immediate sensory modality and linked to salient behavioral events.

INTRODUCTION

Apical dendrites are a common feature of pyramidal neurons throughout the mammalian neocortex, suggesting a general function in cortical computation. Pyramidal neurons in cortical layers 2/3 and 5 send apical dendrites to the surface of the cortex, where they arborize in layer 1. Layer 1 is composed almost entirely of these apical dendrites and axons from both local and distant sources. Being devoid of somata except for a sparse population of inhibitory cells, layer 1 has been largely inaccessible by electrophysiology during behavior. Consequently, the role of apical dendrites in cortical processing remains mysterious.

In the whisker representation of rodent primary somatosensory cortex ("barrel cortex"), long-range axons from diverse areas, including motor cortex, secondary somatosensory cortex, and secondary somatosensory thalamus, synapse extensively onto these apical tufts (Petreanu et al., 2009; Wimmer et al., 2010), potentially modulating sensory processing in this region. Distal synapses onto tufts can engage potent active conductances that generate dendritic calcium spikes, which can boost the response of a pyramidal cell to ascending sensory input onto its basal dendrites, as well as potentiate synaptic connections onto the tuft (Cichon and Gan, 2015; Gambino et al., 2014; Larkum, 2013; Waters et al., 2003; Xu et al., 2012). The apical tuft may therefore be a key site for learning associations among multiple sensory and behavioral representations in the brain.

The only previously identified triggers of global apical tuft dendritic spikes are the same events that drive strong somatic spiking. Sensory stimuli are effective triggers of apical dendrites in primary sensory cortex, limb movement in the case of primary motor cortex, and location within an environment in the case of the hippocampus (Cichon and Gan, 2015; Murayama et al., 2009; Sheffield and Dombeck, 2015; Xu et al., 2012). Here, we demonstrate that learned associations reinforced by reward can become potent additional drivers of apical dendrite activity, suggesting that apical dendrites could be a major conduit for assimilating disparate non-modality-specific, contextual information into a sensory representation. We recorded calcium signals in apical dendrites and somata of cortical neurons using 2-photon imaging of sensors genetically targeted to specific layers of the barrel cortex while mice performed a tactile detection task. Reward-related signals were prominent in the apicals of layers 2/3 and 5 pyramidal neurons within layer 1 but not in the somata of layer 4 neurons, indicative of a non-afferent origin. These reward-reinforced associations emerged with learning and were task specific, in that such signals in the barrel cortex required training on a whisker-based task. Our results suggest that modality-specific reinforcement recruits layer 1 apical dendrites of primary sensory cortex into new representations that extend beyond their normal repertoire of environmental sensory stimuli.

 © 2019 The Author(s).
This is an open access article under the CC BY license (http://creativecommons.org/licenses/by/4.0/).

RESULTS

To examine the activity of apical dendrites within layer 1 during behavior, we first combined a transgenic mouse line expressing Cre recombinase specifically in layer 5 pyramidal neurons (Rbp4-Cre) with viral expression of the genetically encoded calcium indicator GCaMP6f (Chen et al., 2013). This technique labeled ~45% of layer 5 neurons (37/82 GCaMP6f$^+$/NeuN$^+$ cells), including their apical tuft dendrites in layer 1, without expression in the pyramidal cells of other layers (Figure 1A). This population includes diverse subtypes of layer 5 pyramidal neurons, including corticocortical and corticofugal cells (Gerfen et al., 2013). Water-restricted mice were trained on a head-fixed whisker-based pole detection task (Figure 1B). Mice were required to release a lever, when presented with a pole, to obtain a water reward (pole/"Go" trials) and withhold responses when the pole moved in the opposite direction and beyond the reach of the animal's whiskers (catch/"NoGo" trials), which additionally controls for auditory stimuli. After mapping the somatotopy of the barrel cortex by intrinsic signal imaging (Figure 1C), we used 2-photon microscopy during the behavior to monitor the calcium activity in numerous single apical tuft dendrites in cortical locations corresponding to whiskers contacting the pole (Figure 1D). We observed calcium events in the dendritic structures of substantial spatial extent, consistent with global, tuft-wide voltage-gated calcium spikes rather than localized *N*-methyl-D-aspartate (NMDA) receptor-mediated spikes. These calcium events could additionally reflect backpropagating somatic action potentials.

We first averaged fluorescence over the imaged region to assess overall population activity in apical tufts of layer 5 pyramidal neurons (44 sessions in 4 mice). Within a behavioral session, single-trial fluorescence showed a prominent short-latency peak (950 ± 37 ms after trial start, 580 ± 42 ms after first whisker contact) immediately following the presentation of the pole (Figure 1E), which previous studies have demonstrated is due to active contacts of the whisker against the pole (Manita et al., 2015; Xu et al., 2012). We also noticed a second peak at a longer latency, both in single correct Go trials and the group averages (Figures 1F, blue, and S1). Neither peak was present in correct NoGo trials, in which the pole was absent (Figure 1F, red).

Neurons in the primary visual cortex are sensitive to reward timing (Shuler and Bear, 2006), and we suspected that the long-latency peaks in apical tufts may be driven by behavioral feedback, such as rewards. Randomly varying the delay between correct responses to Go trials and water reward administration by 0, 250, or 500 ms shifted the second peak correspondingly, whereas the initial short-latency peak was invariant (single session in Figure 1G, group average in Figure 1H). Seventy-five percent of sessions (33/44) exhibited a discernible second peak, but noise and variability may have masked second peaks in the other sessions. Therefore, we analyzed sessions by calculating second peak latencies for each reward delay within each session. Second peak latency followed the reward delay times (Figure 2A). We regressed second peak latency against reward delay, which was significantly related across all of the sessions ($p < 10^{-4}$, n = 33 sessions in 4 mice).

To further examine the effects of reward on apical dendritic activity in the absence of active contacts, we randomly administered water rewards during a small percentage of the inter-trial intervals (ITIs). Unexpected random rewards during inter-trial intervals elicited a calcium influx of qualitatively similar timing and amplitude to the long-latency peak during trials (Figure 2B). These data indicate that rewards can influence the activity of apical tufts in the primary sensory cortex in diverse behavioral epochs. In addition, dendritic activity during this ITI period when the pole is absent indicates that these calcium events can occur independently of whisker contacts.

Apical activity during rewards could result from motor inputs into layer 1 (Petreanu et al., 2012). Reward consumption inherently involves licking, but isolated spontaneous licking bouts during inter-trial intervals in the absence of water did not increase calcium to the level seen with random rewards (Figure 2C; $p < 0.001$, n = 44 sessions from 4 mice). Another possibility is that reward delivery could arouse a mouse and induce additional whisking. Whisking did not appear to consistently drive calcium influx (Figure S2), but a detailed analysis revealed a weak correlation of whisking and calcium (Figure S3). This correlation, however, fell to nearly 0 in those epochs in which rewards were administered (Figures S3B and S3D). As with licking, calcium responses to the onset of isolated whisking bouts differed markedly from responses to unexpected isolated rewards in the same sessions (Figure 2D; p = 0.0193, n = 6 sessions from 2 mice), suggesting that motor input cannot explain our results.

Before they were trained on pole detection, the mice were pretrained for 1 week to press a lever for a water reward while freely moving and then for 2 to 3 additional weeks while head fixed. Mice were imaged during the last 2 days of head-fixed pretraining and were therefore highly habituated at the time of imaging. Water rewards given to mice that were proficient at the lever-pressing task did not elicit dendritic calcium (Figure 2E), in contrast to random rewards given during the detection task (p = 0.0035, n = 11 lever-task sessions from 3 of the 4 mice used in the 44 detection task sessions). This difference in calcium influx cannot be explained by a difference in licking, as both tasks cause licking to begin to increase 300–400 ms after the lever response and to peak at ~5.5 licks per second at ~1 s after the response. Again, long-latency dendritic activity cannot be explained by reward-triggering simple motor patterns, such as licking and swallowing, or sensations, such as tactile and gustatory stimulation of the tongue. In addition, this result indicates that reward-associated activity in apical dendrites does not reflect a global reward signal but rather task specificity, such that the behavior must engage the specific cortical region in question. Dendritic responses to isolated rewards during the pole detection task slowly increased during learning (Figure 2F; linear regression, p = 0.0055, n = 4 animals). Similarly, the long-latency peak during task trials grew with training (Figure S4). These results point to the emergence of a learned association between whisker-related neural activity and synaptic inputs linked to reward receipt.

Since average responses to trial and random rewards are similar in amplitude, they could represent the activity of a single population of reward-sensitive dendrites or, alternatively, activity in distinct populations. To assess the activity of individual apical

OPEN
ACCESS
CellPress

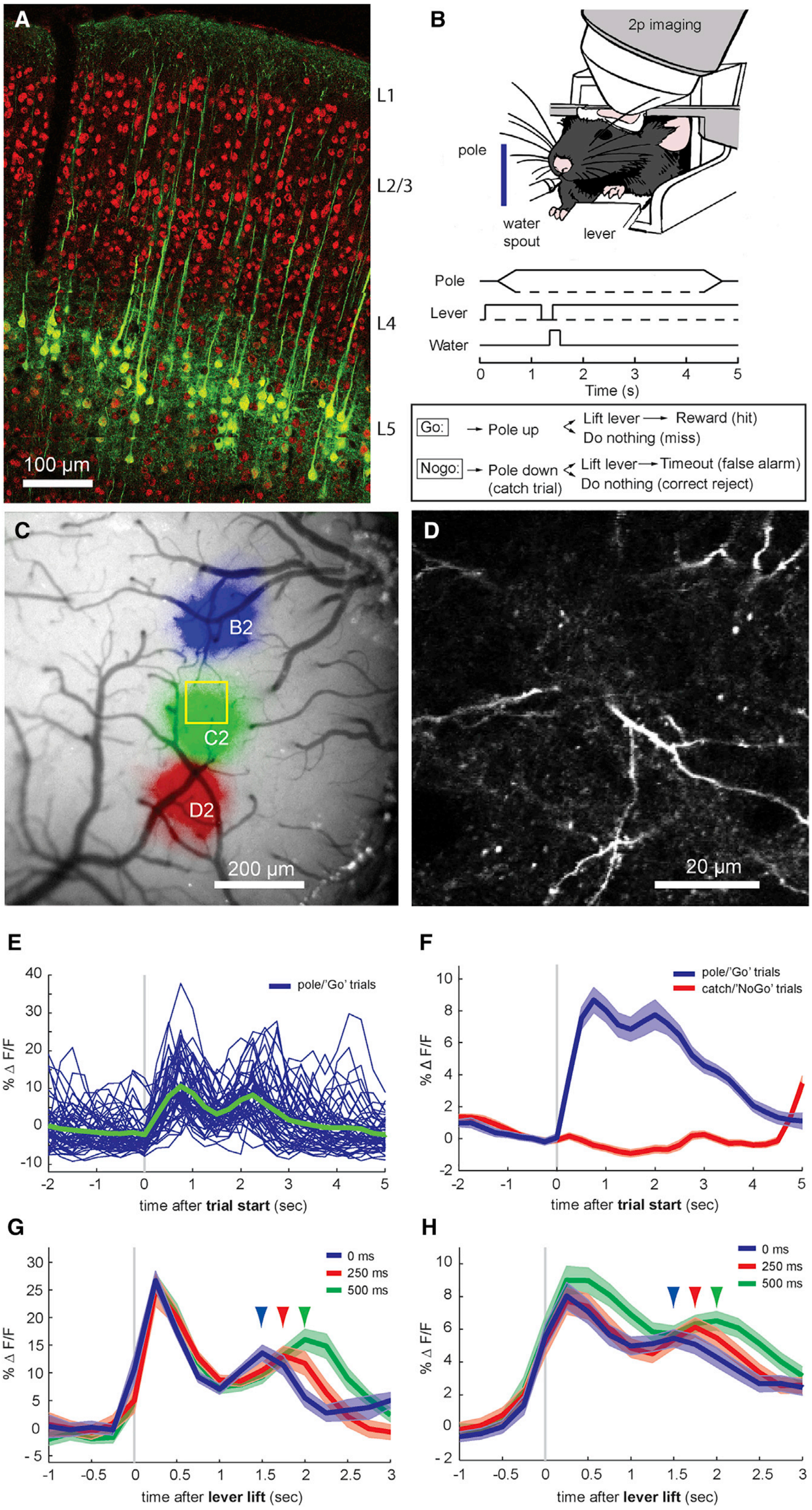

Figure 1. Imaging Calcium in Layer 5 Apical Dendrites during a Whisker-Based Pole Detection Task

(A) Labeling of layer 5 pyramidal neurons in mouse barrel cortex with the Rbp4-Cre mouse transgenic line. Green: GCaMP6 expression after injection of an Rbp4-Cre mouse with AAV9-hSyn-FLEX-GCaMP6f. Red: immunohistochemical labeling for NeuN.

(B) Behavioral 2-photon imaging setup of a mouse performing a whisker-based pole detection task. A head-fixed mouse presses a lever, at which time a pole moves either into the animal's whisker field (pole/Go trials) or a similar distance away from the whiskers (catch/NoGo trials). The animal then must lift its paw from the lever to indicate the presence of the pole (Go trials) or withhold lever lifting for the 3-s duration of the trial (NoGo trials). Correct Go responses are rewarded with a drop of water and a 3-s drinking period, while incorrect NoGo responses are punished with an 8-s timeout period, after which time the pole moves back to its starting position.

(C) Intrinsic optical signal mapping of the mouse barrel cortex during single-whisker stimulation. Colors indicate the regions that are active in response to the repetitive stimulation of D2 (red), C2 (green), or B2 (blue) whiskers.

(D) Single-frame GCaMP6f fluorescence from a layer 5 pyramidal neuron apical dendrite within layer 1 during *in vivo* 2-photon calcium imaging (depth 60 µm).

(E) Whole-frame layer 1 GCaMP6f fluorescence for each correct pole/Go trial from a single session during detection task performance and average (green, n = 93 trials).

(F) Averages for correct pole/Go trials and catch/NoGo trials (n = 44 sessions from 4 mice). Shaded areas, SEMs.

(G) Average for a single session with varying delay between lever lift and reward. Arrows indicate long-latency peak times for 0-, 250-, or 500-ms delays after response (n = 86 trials).

(H) Average from Rbp4 animals (n = 44 sessions from 4 mice).

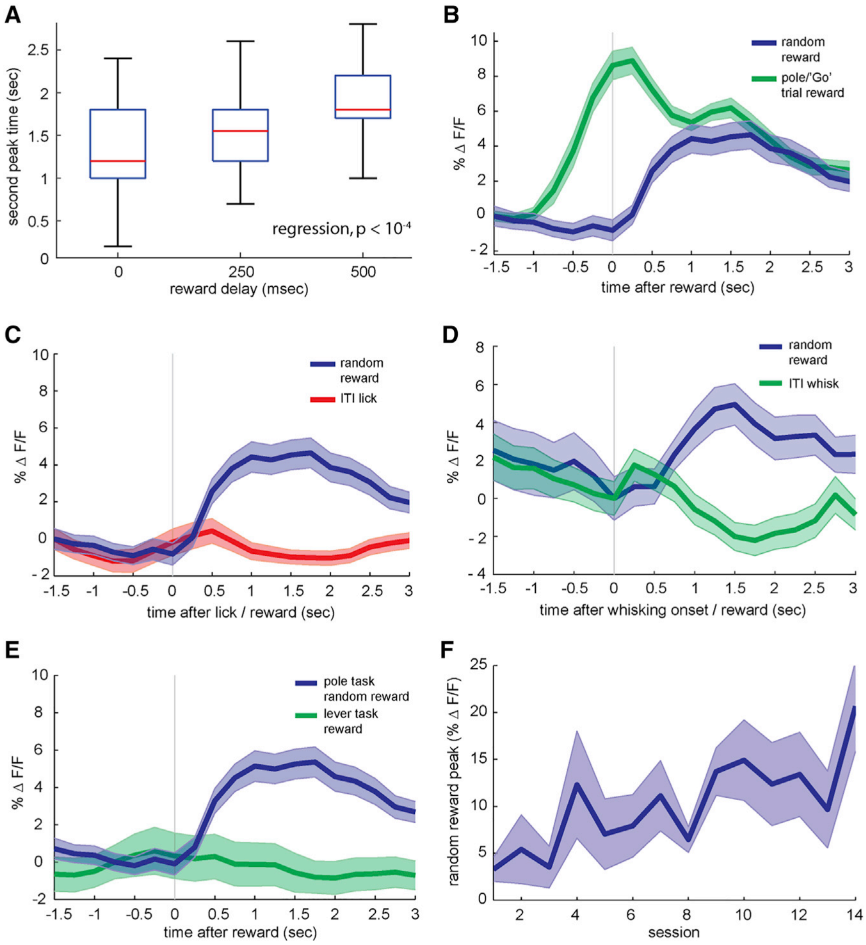

Figure 2. Random Rewards and Motor Inputs

(A) Timing of late-phase calcium peak during correct pole trials for rewards given at 0, 250, or 500 ms after the behavioral response (n = 33 sessions from 4 animals, $p < 10^{-4}$).

(B) Blue: GCaMP fluorescence recorded in layer 1 from Rbp4/GCaMP6 animals in response to rewards given randomly during inter-trial intervals (ITIs) (n = 44 sessions from 4 animals). Green: average whole-frame GCaMP6f fluorescence during correct rewarded stimuli. Shaded areas, SEMs.

(C) Average whole-frame fluorescence in response to random inter-trial interval rewards compared with isolated licking bouts. Blue: random rewards, green: spontaneous licking bouts during inter-trial intervals (n = 44 sessions from 4 animals).

(D) Whole-frame fluorescence in layer 1 triggered on spontaneous whisking bouts during inter-trial intervals. Green: inter-trial interval whisking bout GCaMP6 fluorescence (n = 88 whisking bouts from 2 animals), blue: random inter-trial interval reward responses (n = 46 rewards from the same 2 animals).

(E) Calcium response to random inter-trial interval rewards, as above, compared with rewards given during lever pretraining, in which a mouse simply presses a lever to receive a water reward (n = 11 sessions from 3 mice).

(F) Growth of random inter-trial interval reward peak response across sessions (n = 4 mice, p = 0.0055).

dendrites activated at distinct times during the task, we segmented movies using a sparse non-negative matrix factorization method that forms an overlapping clustering of pixels according to their temporal covariance (Pnevmatikakis et al., 2016). Thus, while pixels within a factor (segmented dendrite) necessarily have correlated activity, different factors (different segmented dendrites) may be active at different times. This method yielded 18–72 (mean of 38) putative single dendritic arbors for each movie. Some dendrites were active selectively during trials (Figure 3A, bottom) while others were active during inter-trial intervals and silent during trials (Figure 3A, top). Individual segmented dendrites were substantial in their spatial extent (>100 μm), which is consistent with global voltage-gated calcium spikes and backpropagating action potentials.

As a population, individual dendritic arbors had peak activity times that tiled both pre-trial and trial epochs (Figure 3B; n = 22 sessions in 3 Rbp4 mice). We observed a wide continuum of dendrites preferentially active in the pre-trial versus trial epochs (Figure 3C). A subset of dendrites (48/530, 9% of total) were activated by random rewards during inter-trial intervals (Figure 3C, red), and these were more likely to be the dendrites that were suppressed during the trial (25/167 versus 23/363, $p < 0.001$; Figures 3C and 3D). In contrast, dendrites with enhanced activity during trial epochs were more likely to track reward timing, as in Figures 1G and 1H (Figures 3C and 3D, green; 52/363 versus 11/167, p = 0.01). Furthermore, reward-tracking dendrites tended to respond to the initial contact of the whiskers against the pole (Figure 3E), unlike random reward-selective dendrites (Figures 3F and 3H). The large subset of dendrites that were not reward selective appeared to have contact activity like reward-tracking dendrites (Figure 3G). None of these subsets of dendrites exhibited strong lick modulation (Figure 3I). Thus, the effects of reward within trials and unanticipated rewards on the population of apical dendrites in the barrel cortex (Figures 1 and 2) are not found in every neuron, but in fact reflect different subsets of cells. Furthermore, these subsets of cells are natively active during the behavioral epoch in which the reward is given.

Like layer 5 cells, layer 2/3 pyramidal neurons also extend their apical dendrites into layer 1. To examine whether reward similarly influenced layer 2/3 neurons, we used another transgenic mouse line to selectively express GCaMP6f in this layer (Cux2-Cre; Figure 4A, center). We imaged calcium activity in the somata of both neurons in layer 2/3 (left) and their apical dendrite tufts in layer 1 (right) in the same mice in paired sessions on the same day. Similar to layer 5 apical dendrites, average calcium signals in the apical tufts of layer 2/3 neurons exhibited a 2-peak structure (Figure 4B, blue), indicating that reward-associated dendritic activity is not unique to layer 5

OPEN ACCESS
CellPress

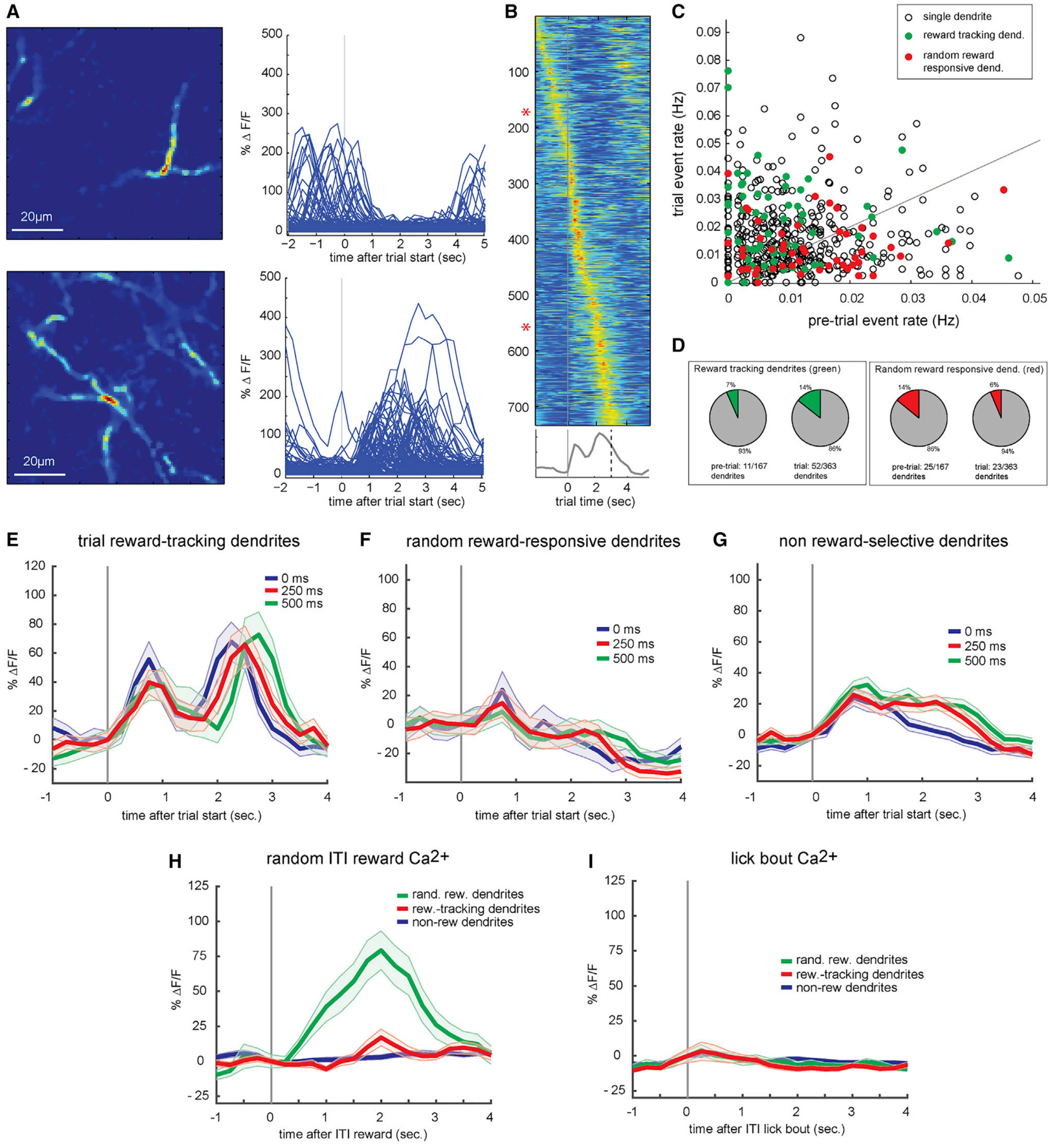

Figure 3. Firing Properties of Single-Layer 5 Tuft Dendrites

(A) Spatial profiles and single-trial calcium responses of 2 putative single dendrites from the same behavioral session. Top: a single dendrite that responds preferentially during inter-trial intervals preceding a trial and is suppressed during the trial. Bottom: single dendrite that responds during the trial epoch.

(B) Average calcium responses for 733 putative single dendritic arbors, sorted by time of peak response with respect to trial initiation. Asterisks indicate dendrites shown in (A).

(C) Single dendrites that respond preferentially during random inter-trial interval rewards (red) and pole trial reward-tracking dendrites (green) plotted based upon average response rate before and during pole trials (n = 530).

(D) Proportions of dendrites active before trial initiation (left) and during the trial (right) responding to either trial rewards (green) or random rewards (red).

(legend continued on next page)

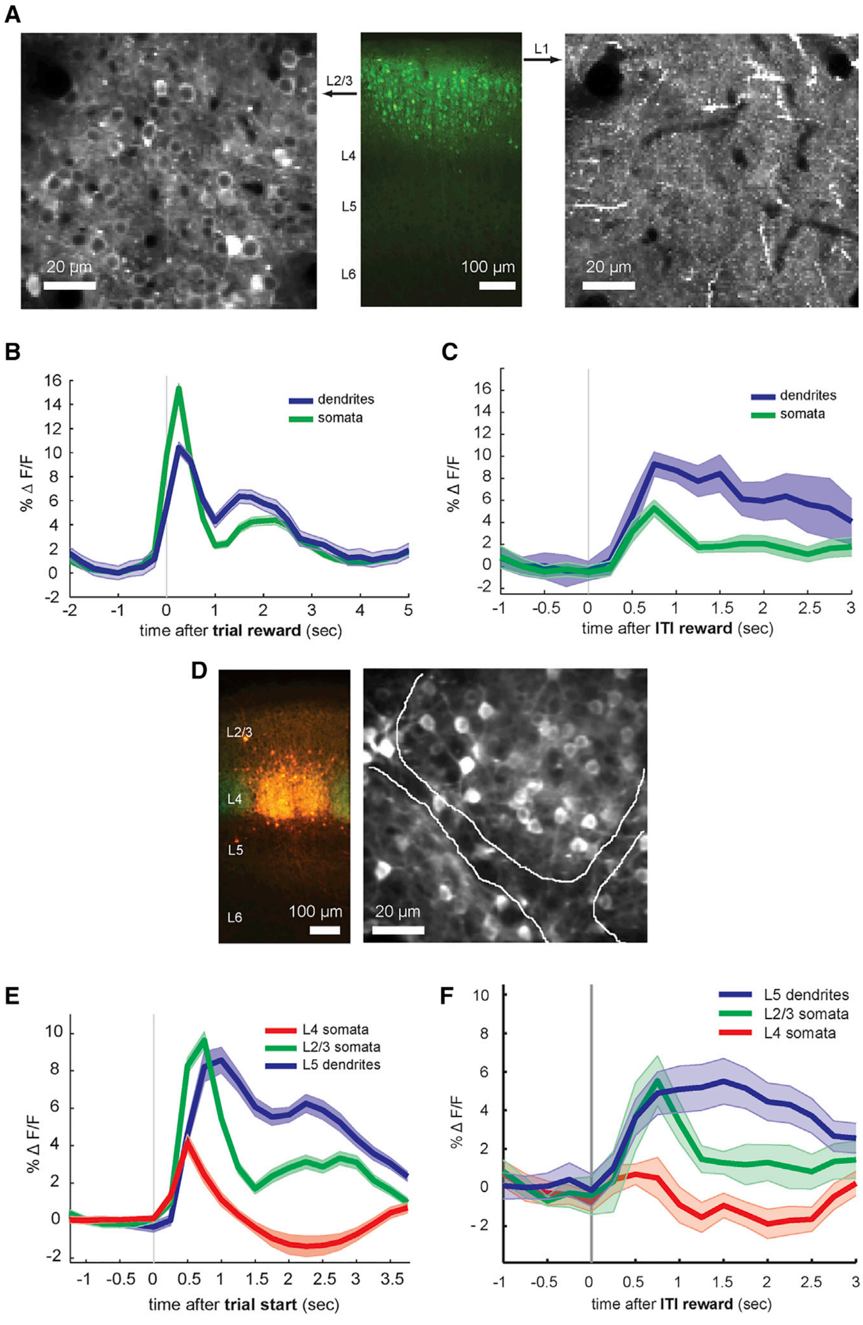

Figure 4. Layer-Specific Reward-Related Responses

(A) Labeling layer 2/3 pyramidal neurons in the mouse barrel cortex with the Cux2-Cre transgenic line. Center: GCaMP6f fluorescence in mouse brain slice from a Cux2-Cre mouse injected with Cre-inducible GCaMP6 virus into the barrel cortex. Left: single 2-photon imaging plane from layer 2/3 somatic layer (approximately 200 μm deep from pial surface) *in vivo*. Right: corresponding imaging plane in layer 1 (approximately 40 μm deep) in the same location.

(B) Average whole-frame fluorescence for correct pole trials in Cux2-Cre/GCaMP6f animals, from either layer 1 ("dendrites") or layer 2/3 ("somata") recorded consecutively in the same animal and barrel region (n = 6 sessions each from 2 animals). Shaded areas, SEMs.

(C) Average calcium signals in layer 2/3 dendritic and somatic regions in response to random inter-trial interval rewards, in same sessions as (B).

(D) Labeling of layer 4 neurons with the Nr5a1-Cre transgenic line. Left: AAV1-CAG-FLEX-tdTomato injected into the barrel cortex of an Nr5a1-Cre/ROSA-GFP mouse. Red: viral tdTomato expression, green: transgenic GFP expression. Right: single-plane 2-photon image of GCaMP6f expression *in vivo* in layer 4 of an Nr5a1-Cre mouse injected with Cre-inducible GCaMP6f virus, showing the approximate boundaries between 2 adjacent barrels.

(E) Comparison of average whole-frame fluorescence in different cortical layers during task performance. Red: pole/Go trial responses from layer 4 somata (n = 12 sessions from 3 mice). Green: layer 2/3 somata (n = 6 sessions from 2 mice). Blue: layer 5 apical tufts (n = 44 sessions from 4 mice).

(F) Random inter-trial interval reward responses in different cortical layers. Red: layer 4 somata, green: layer 2/3 somata, blue: layer 5 dendrites (same data as Figure 5E).

pyramidal cells. Moreover, the same pattern was observed in layer 2/3 somata (Figure 4B, green), albeit to a lesser extent, suggesting the generation of somatic action potentials in the same period. This observation is consistent with *in vivo* demonstrations that layer 2/3 apical dendrite activity strongly depolarizes the soma (Palmer et al., 2014). Relative to the short-latency calcium peak, the long-latency peak was larger in frame averages of layer 2/3 apicals than their somata (Figure 4B), and reward tracking was observable for layer 2/3 somata and dendrites (Figure S5). Unexpected rewards elicited calcium transients in both compartments of layer 2/3 cells (Figure 4C),

(E) Average calcium responses of trial reward-tracking dendrites (n = 63) to rewards delayed by 0 (blue), 250 (red), or 500 ms after lever lift (green). Shaded areas, SEM.
(F) Same as (E), but for unexpected inter-trial interval reward-responsive dendrites (n = 48).
(G) Same as (E), but for reward-unresponsive dendrites (n = 419).
(H) Responses of trial reward-tracking (green), unexpected inter-trial interval reward-responsive (red), and non-reward-responsive (blue) dendrites to unexpected inter-trial interval rewards.
(I) Same as (H), but for spontaneous inter-trial interval licking bouts.

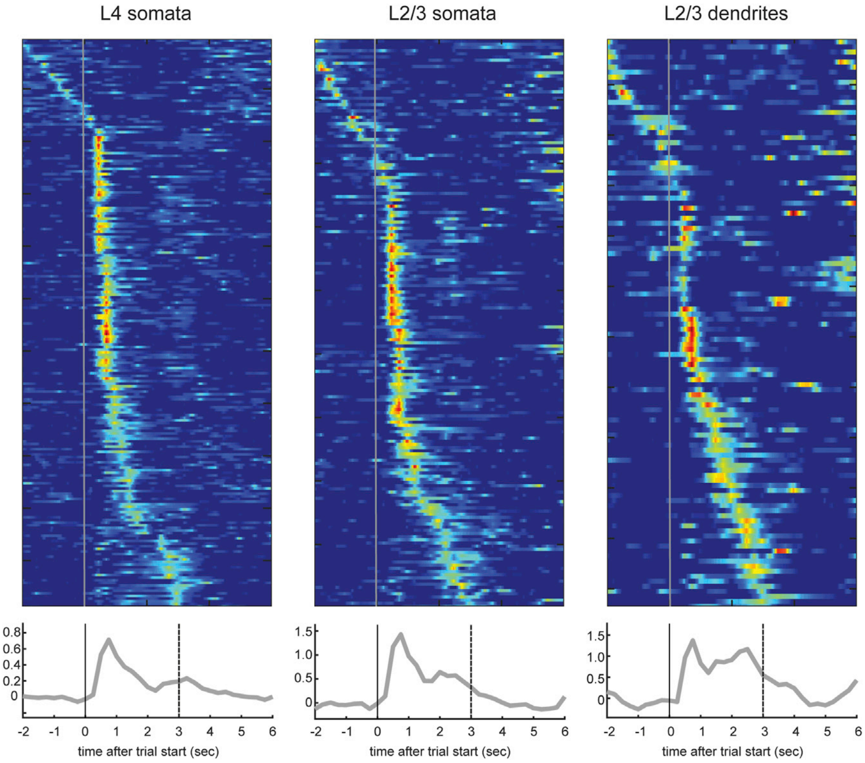

Figure 5. Activity of Individual Somata and Dendrites in Layers 2/3 and 4

Top: trial-averaged calcium responses during hit trials from layer (L) 4 somata (n = 217 somata from 6 sessions in 3 mice), layer 2/3 somata (n = 179 somata from 4 sessions in 2 mice), and layer 2/3 dendrites (n = 113 dendrites). Layer 2/3 somata and dendrites were recorded on the same day, from the same somatotopic location in the same animal. Bottom: average calcium responses in percentage of dF/F from all of the dendrites from these animals.

and the relative magnitudes of these transients were similar to long-latency peaks during trials.

Excitatory layer 4 cells (labeled by Nr5a1-Cre), which lack apical dendrites reaching layer 1, showed average activity only during the initial whisker contact period, at a time corresponding to the first peak in the layer 2/3 and layer 5 responses (Figures 4D and 4E). Similarly, unexpected isolated rewards did not increase calcium activity in layer 4 (Figure 4F). Analysis of individual somata and dendrites showed that most layer 4 somata respond more strongly to contacts than trial rewards (Figure 5, left). However, a subset of layer 4 somata exhibited trial reward period activity, albeit more weakly and in smaller number than for contacts, possibly reflecting signals entering from pyramidal cell layers onto the short layer 4 star pyramid apicals, potential misclassification of some deep layer 2/3 cells as layer 4 when using this transgenic line, and/or possible exaggeration of the second peak by our segmentation method (non-negative matrix factorization), which can underestimate strong negativity, like that in Figure 4E.

Activity in individual layer 2/3 apical dendrites and somata (Figure 5, center and right) was consistent with population signals (Figure 4). Reward periods engender progressively stronger activity relative to contacts in individual layer 2/3 somata (Figure 5, center), then layer 2/3 dendrites (right), and finally layer 5 dendrites (Figure 3B). Moreover, any reward period activity in layer 4 somata followed rather than preceded reward period activity in layer 5 dendrites and layer 2/3 somata and dendrites (Figure 5). Thus, the long-latency reward-associated peak is unlikely to be explained by sensory afference (whisker contact signals) transmitted through layer 4 and suggests another pathway, potentially involving apical dendrites, where the signals are more pronounced.

DISCUSSION

Using mouse transgenic lines to target calcium indicators to specific layers, we demonstrated that apical dendrites can incorporate non-modality-specific information into sensory representations. For mice experienced at an operant whisker-based task, an event involving no immediate whisker contact whatsoever (delivery of a reward water droplet) elicited pronounced long-latency dendritic spiking (and somatic output) in the barrel cortex. In retrospect, such a secondary peak is visible in previous imaging of layer 5 apical dendrites during a similar task (Xu et al., 2012). Our stochastic manipulation of reward administration allowed us to dissociate the coupling of sensory input, response, and reinforcement and reveals a unique relationship of rewarded events to apical activity.

Any of the many areas synapsing on apical dendrites of barrel cortex neurons—for example, motor cortex, secondary somatosensory cortex, secondary somatosensory thalamus (POm)—could be a trigger for these apical dendritic events. Even the apical dendrites that were not locked to an obvious behavioral event (e.g., whisker contact, lever press, reward) appeared to have a preferred time of activity within or around a trial (Figure 3), perhaps reflecting phasic locking to the activity of an ensemble elsewhere in the brain. These ensembles may therefore encode information other than whisker contacts, such as other sensory modalities or internal knowledge (e.g., motor efference copy, task structure, expectations, object identity) that may be important in predictive coding and appropriate responses with respect to modality-specific sensory input for salient stimuli. Potentiation of these synapses through reinforcement may solidify their ability to elicit a global dendritic spike, thereby simultaneously altering somatic spiking in the barrel cortex and the synapse's potential for future plasticity. Additional studies are needed to investigate the degree to which apical recruitment may also reflect the enhanced generation and backpropagation of somatic spikes.

Although dopamine is often implicated in reinforcement effects on neural circuits, dopaminergic terminals are relatively sparse in primary sensory areas. Neurons in rodent primary visual cortex, however, have been found to be sensitive to reward timing during

operant tasks, an effect that is mediated by a cholinergic-dependent mechanism (Chubykin et al., 2013; Shuler and Bear, 2006). Acetylcholine disinhibits apical dendrites by suppressing particular subpopulations of layer 1 interneurons (Brombas et al., 2014). Furthermore, similar layer 1 interneurons in auditory and prefrontal cortices, as well as cholinergic basal forebrain neurons, are activated by whisking, rewards, and punishments, leading to the inhibition of apical dendrite-targeting interneurons (Eggermann et al., 2014; Hangya et al., 2015; Letzkus et al., 2011; Pi et al., 2013). Thus, salient events such as behavioral reinforcement during active sensing could lead to the disinhibition of apical dendrites, which may promote the generation of tuft calcium spikes when they coincide with apical synaptic inputs. This suggests that cortical pyramidal neurons with dendrites in layer 1 could learn new associations through the plasticity of apical inputs, which is modulated by the cholinergic disinhibition of apical dendrites. Norepinephrine is another important candidate needing further study in this context (Labarrera et al., 2018).

In the motor cortex, reward is able to potentiate the somatic discharges of weakly active cells when paired with the firing of the cell (Hira et al., 2014). Similarly, our findings that dendrites active during inter-trial intervals are preferentially triggered by unexpected isolated rewards and that dendrites active during trials track reward timing suggest a mechanism by which subsets of cells could come to be recruited into ensembles encoding temporally specific contextual information through reinforcement. Learning has been shown to enhance the responses of visual cortex neurons to non-sensory factors such as task outcome, in addition to sensory features such as stimulus orientation (Poort et al., 2015). Recent imaging work in the auditory system has concluded that the cholinergic modulation of inhibition may play a role in the processing of contextual information (Kuchibhotla et al., 2017).

Our method for segmenting individual dendrites based on spatiotemporal covariance (Pnevmatikakis et al., 2016) does not discount the possibility that two putative dendritic trees belong to the same neuron. In addition, it is possible that some putative dendritic trees are in fact two highly synchronized neurons. Even if this were true, our study demonstrates phenomena related to reward-learned associations in a primary sensory cortex and a higher level of neuronal specificity than expected from a purely global neuromodulatory signal. Further studies are needed to assess the degree to which learned responses to unexpected rewards and trial-related rewards are cell type specific versus branch specific.

Our study suggests that salient behavioral events, such as rewards, can modify the occurrence of apical tuft spikes, presumably through the plasticity of long-range connections encoding context. This could afford a powerful generalized mechanism for encoding task-relevant information to any given cortical area, including associations with multiple sensory modalities and motor behaviors, as well as predictions about upcoming inputs.

STAR★METHODS

Detailed methods are provided in the online version of this paper and include the following:

- KEY RESOURCES TABLE
- CONTACT FOR REAGENT AND RESOURCE SHARING
- EXPERIMENTAL MODEL AND SUBJECT DETAILS
- METHOD DETAILS
  - Behavior
  - Surgery
  - Imaging
- QUANTIFICATION AND STATISTICAL ANALYSIS
- DATA AND SOFTWARE AVAILABILITY

SUPPLEMENTAL INFORMATION

Supplemental Information can be found with this article online at https://doi.org/10.1016/j.celrep.2019.01.093.

ACKNOWLEDGMENTS

We thank Bernd Kuhn for advice on imaging; Bina Santoro and Y. Kate Hong for assistance with transgenic mouse lines; Chris Rodgers for help with whisker tracking; Patrick Kaifosh for assistance with imaging data motion correction; Adele Sung, Isabel Stillman, Vangie Shue, Tim Michaels, and Ronald Nicholson for pilot behavioral experiments; Douglas Repetto for help developing the OpenMaze behavioral system; and Nouri Zander for an illustration. We thank Attila Losonczy, Nate Sawtell, and Michael Shadlen for comments on the manuscript. Funding provided by the Dana Foundation Brain and Immuno-imaging program, the Klingenstein Fund, the Ludwig Schaefer Fund, the Rita Allen Foundation, the Sanford Grossman-Fred Kavli Faculty Scholar program, NINDS (R01 NS069679 and R01 NS094659), NIBIB (R01 EB22913), and NIMH (TG MH015174).

AUTHOR CONTRIBUTIONS

C.O.L. and R.M.B. conceived the project. C.O.L. constructed the behavioral apparatus, trained the animals, and performed the imaging. R.M.B. constructed the intrinsic and two-photon imaging setups. E.A.P. and L.P. developed tools for analyzing single dendrites. C.O.L. and R.M.B. analyzed the data and wrote the paper.

DECLARATION OF INTERESTS

The authors declare no competing interests.

Received: February 8, 2016
Revised: September 27, 2018
Accepted: January 24, 2019
Published: February 19, 2019

REFERENCES

Brombas, A., Fletcher, L.N., and Williams, S.R. (2014). Activity-dependent modulation of layer 1 inhibitory neocortical circuits by acetylcholine. J. Neurosci. *34*, 1932–1941.

Chen, T.W., Wardill, T.J., Sun, Y., Pulver, S.R., Renninger, S.L., Baohan, A., Schreiter, E.R., Kerr, R.A., Orger, M.B., Jayaraman, V., et al. (2013). Ultrasensitive fluorescent proteins for imaging neuronal activity. Nature *499*, 295–300.

Chubykin, A.A., Roach, E.B., Bear, M.F., and Shuler, M.G. (2013). A cholinergic mechanism for reward timing within primary visual cortex. Neuron *77*, 723–735.

Cichon, J., and Gan, W.B. (2015). Branch-specific dendritic Ca(2+) spikes cause persistent synaptic plasticity. Nature *520*, 180–185.

Clack, N.G., O'Connor, D.H., Huber, D., Petreanu, L., Hires, A., Peron, S., Svoboda, K., and Myers, E.W. (2012). Automated tracking of whiskers in videos of head fixed rodents. PLoS Comput. Biol. *8*, e1002591.

Eggermann, E., Kremer, Y., Crochet, S., and Petersen, C.C.H. (2014). Cholinergic signals in mouse barrel cortex during active whisker sensing. Cell Rep. *9*, 1654–1660.

Franco, S.J., Gil-Sanz, C., Martinez-Garay, I., Espinosa, A., Harkins-Perry, S.R., Ramos, C., and Müller, U. (2012). Fate-restricted neural progenitors in the mammalian cerebral cortex. Science *337*, 746–749.

Gambino, F., Pagès, S., Kehayas, V., Baptista, D., Tatti, R., Carleton, A., and Holtmaat, A. (2014). Sensory-evoked LTP driven by dendritic plateau potentials in vivo. Nature *515*, 116–119.

Gerfen, C.R., Paletzki, R., and Heintz, N. (2013). GENSAT BAC cre-recombinase driver lines to study the functional organization of cerebral cortical and basal ganglia circuits. Neuron *80*, 1368–1383.

Hangya, B., Ranade, S.P., Lorenc, M., and Kepecs, A. (2015). Central Cholinergic Neurons Are Rapidly Recruited by Reinforcement Feedback. Cell *162*, 1155–1168.

Hira, R., Ohkubo, F., Masamizu, Y., Ohkura, M., Nakai, J., Okada, T., and Matsuzaki, M. (2014). Reward-timing-dependent bidirectional modulation of cortical microcircuits during optical single-neuron operant conditioning. Nat. Commun. *5*, 5551.

Kaifosh, P., Zaremba, J.D., Danielson, N.B., and Losonczy, A. (2014). SIMA: Python software for analysis of dynamic fluorescence imaging data. Front. Neuroinform. *8*, 80.

Kuchibhotla, K.V., Gill, J.V., Lindsay, G.W., Papadoyannis, E.S., Field, R.E., Sten, T.A., Miller, K.D., and Froemke, R.C. (2017). Parallel processing by cortical inhibition enables context-dependent behavior. Nat. Neurosci. *20*, 62–71.

Labarrera, C., Deitcher, Y., Dudai, A., Weiner, B., Kaduri Amichai, A., Zylbermann, N., and London, M. (2018). Adrenergic Modulation Regulates the Dendritic Excitability of Layer 5 Pyramidal Neurons In Vivo. Cell Rep. *23*, 1034–1044.

Larkum, M. (2013). A cellular mechanism for cortical associations: an organizing principle for the cerebral cortex. Trends Neurosci. *36*, 141–151.

Letzkus, J.J., Wolff, S.B., Meyer, E.M., Tovote, P., Courtin, J., Herry, C., and Lüthi, A. (2011). A disinhibitory microcircuit for associative fear learning in the auditory cortex. Nature *480*, 331–335.

Manita, S., Suzuki, T., Homma, C., Matsumoto, T., Odagawa, M., Yamada, K., Ota, K., Matsubara, C., Inutsuka, A., Sato, M., et al. (2015). A Top-Down Cortical Circuit for Accurate Sensory Perception. Neuron *86*, 1304–1316.

Murayama, M., Pérez-Garci, E., Nevian, T., Bock, T., Senn, W., and Larkum, M.E. (2009). Dendritic encoding of sensory stimuli controlled by deep cortical interneurons. Nature *457*, 1137–1141.

Palmer, L.M., Shai, A.S., Reeve, J.E., Anderson, H.L., Paulsen, O., and Larkum, M.E. (2014). NMDA spikes enhance action potential generation during sensory input. Nat. Neurosci. *17*, 383–390.

Petreanu, L., Mao, T., Sternson, S.M., and Svoboda, K. (2009). The subcellular organization of neocortical excitatory connections. Nature *457*, 1142–1145.

Petreanu, L., Gutnisky, D.A., Huber, D., Xu, N.L., O'Connor, D.H., Tian, L., Looger, L., and Svoboda, K. (2012). Activity in motor-sensory projections reveals distributed coding in somatosensation. Nature *489*, 299–303.

Pi, H.J., Hangya, B., Kvitsiani, D., Sanders, J.I., Huang, Z.J., and Kepecs, A. (2013). Cortical interneurons that specialize in disinhibitory control. Nature *503*, 521–524.

Pnevmatikakis, E.A., Soudry, D., Gao, Y., Machado, T.A., Merel, J., Pfau, D., Reardon, T., Mu, Y., Lacefield, C., Yang, W., et al. (2016). Simultaneous denoising, deconvolution, and demixing of calcium imaging data. Neuron *89*, 285–299.

Poort, J., Khan, A.G., Pachitariu, M., Nemri, A., Orsolic, I., Krupic, J., Bauza, M., Sahani, M., Keller, G.B., Mrsic-Flogel, T.D., and Hofer, S.B. (2015). Learning Enhances Sensory and Multiple Non-sensory Representations in Primary Visual Cortex. Neuron *86*, 1478–1490.

Sheffield, M.E., and Dombeck, D.A. (2015). Calcium transient prevalence across the dendritic arbour predicts place field properties. Nature *517*, 200–204.

Shuler, M.G., and Bear, M.F. (2006). Reward timing in the primary visual cortex. Science *311*, 1606–1609.

Waters, J., Larkum, M., Sakmann, B., and Helmchen, F. (2003). Supralinear Ca2+ influx into dendritic tufts of layer 2/3 neocortical pyramidal neurons in vitro and in vivo. J. Neurosci. *23*, 8558–8567.

Wimmer, V.C., Bruno, R.M., de Kock, C.P., Kuner, T., and Sakmann, B. (2010). Dimensions of a projection column and architecture of VPM and POm axons in rat vibrissal cortex. Cereb. Cortex *20*, 2265–2276.

Xu, N.L., Harnett, M.T., Williams, S.R., Huber, D., O'Connor, D.H., Svoboda, K., and Magee, J.C. (2012). Nonlinear dendritic integration of sensory and motor input during an active sensing task. Nature *492*, 247–251.

STAR★METHODS

KEY RESOURCES TABLE

REAGENT or RESOURCE	SOURCE	IDENTIFIER
Antibodies		
Mouse anti-NeuN Antibody, clone A60	Millipore	MAB377
Bacterial and Virus Strains		
AAV9.Syn.Flex.GCamP6f.WPRE.SV40	Penn Vector Core	N/A
AAV2.CAG.Flex.tdTomato.WPRE.bGH	Penn Vector Core	N/A
Experimental Models: Organisms/Strains		
Mouse: Rbp4-Cre	GENSAT	Rbp4-Cre_KL100
Mouse: Cux2-Cre	MMRRC	Stock # 032778-MU
Mouse: Nr5a1-Cre	Jackson Laboratories	Stock # 006364
Software and Algorithms		
SIMA	Kaifosh et al., 2014	https://pypi.org/project/sima/
Whisk	Clack et al., 2012	https://www.janelia.org/open-science/whisk-whisker-tracking
Sparse NMF	Pnevmatikakis et al., 2016	https://github.com/flatironinstitute/CaImAn-MATLAB

CONTACT FOR REAGENT AND RESOURCE SHARING

Further information and requests for resources and reagents should be directed to and will be fulfilled by the Lead Contact, Randy M. Bruno (randybruno@columbia.edu).

EXPERIMENTAL MODEL AND SUBJECT DETAILS

To label specific cortical layers, we utilized several mouse transgenic Cre lines: Rbp4-Cre (GENSAT), Cux2-Cre (Franco et al., 2012) (MMRC), and Nr5a1-Cre (Jackson Laboratories). All mice were > 8 wks old and bred as F1 hybrids on a C57B6/129svev background. Both male and females were used. Animals were group housed without enrichment. All procedures were approved by the Institutional Animal Care & Use Committee at Columbia University.

METHOD DETAILS

Behavior

Behavioral experiments were performed with the Arduino-based OpenMaze open-source behavioral system, whose designs are fully described at www.openmaze.org. Prior to training on the tactile detection task, mice were trained to press a lever for a water reward for one week prior to headpost implantation and > 1 week while head-fixed. Lever-trained mice were then either injected with virus (below) or trained to a criterion of 70% correct responses on the tactile detection task before injection. Two weeks after injection, animals were implanted with a chronic cranial window and imaged during the detection task.

The tactile detection task requires that the mouse hold a lever down for > 1 s to initiate a trial, in which a stepper motor moved a small pole (2.15-mm diameter, ~3-4-cm long wooden applicator stick), which started from a position 3-4 cm below the animal. The stepper motor rotated the pole to ~2 mm anterior of the nose and ~10 mm lateral of the nose (pole/Go trials) or in the opposite direction even further away from the whiskers (catch/NoGo trials). Whiskers were not hit passively by the pole during stimulus movement, except in a minority (a few percent) of trials where mice whisked or held their whiskers in a protracted position during pole presentation. Typically, mice initiated whisking after they heard the motor begin to move at the start of the trial. During each trial, the mouse had to lift its paw from the lever within 3 s to indicate the presence of the pole, or keep the lever depressed if the pole was absent. Correct pole/Go trials (“Hits”) were rewarded with a small droplet of water (~8 μl) from a water port, whereas incorrect lever lifts during the catch/NoGo trials (“false alarms”) were punished by an 8-10 s timeout before another trial could be initiated as well as a white noise sound. Imaging during the detection task was from animals that had achieved > 70% correct responses during a previous session. Animals performed 100-200 trials during a behavioral session, which typically lasted 20-30 minutes.

Licks were detected with a capacitance-based touch sensor (Sparkfun). Whisking was monitored with a high-speed imaging camera (at 300 fps with a PhotonFocus CCD camera or at 187 fps with Sony PS3eye camera) and automatically measured offline using published software (Clack et al., 2012). For experiments aimed at dissecting the effect of reward on apical tuft dendrites,

random rewards were given during the inter-trial interval with 2%–5% probability, and pole/Go trial reward administrations were delayed by 0, 250, or 500 ms randomly each trial (0, 250 ms for Cux2-Cre mice).

The overall reward rate, which varied across sessions and mice, was approximately 5-10 rewards/min for the pole task and 10-15 rewards/min for the lever task.

Surgery

Animals were anesthetized with isoflurane and implanted with a light-weight stainless steel headpost embedded in dental acrylic affixed to the mouse's skull after application of a thin layer of Vetbond (3M). Mice recovered for one week before habituation to head fixation. For virus injections, mice were anesthetized with isoflurane and injected with adeno-associated virus (serotypes 1 or 9) encoding the fluorescent calcium indicator GCaMP6f in a Cre recombinase-specific manner. The human synapsin promoter (AAV-hSyn-FLEX-GCaMP6f; Penn Vector Core, GECI consortium) was used for Rbp4 and Cux2 lines, and the CAG promoter for the Nr5a1 line (titers $\sim 2x10^{13}$ cfu/mL). 100 nL of virus was injected at 1:2-8 dilution in ACSF using a pulled pipette (20-30 μm ID) at a depth appropriate for the cortical layer of interest (L5: 800 μm, L2/3: 200 μm, L4: 500 μm), 1.6 mm posterior to bregma and 3.2 mm lateral of the midline. Two-photon imaging was performed ~2 weeks after viral infection. For cranial window implantation (a few days prior to imaging), animals were injected with dexamethasone 1 h prior to surgery, at which time they were anesthetized with isoflurane. A 3-mm hole was drilled in the skull overlying the barrel cortex, and the dura removed from the region of the craniotomy. A 3-mm glass coverslip was inserted into the craniotomy and cemented into place with Vetbond.

Imaging

Cortical regions corresponding to particular whiskers were identified using intrinsic optical signal imaging. Single whiskers in isoflurane anesthetized mice were stimulated at 5 Hz using a piezoelectric bimorph while recording the reflectance of 700-nm incandescent light with a Rolera CCD camera (QImaging) using software custom-written in Labview (National Instruments).

Two-photon imaging was performed using a Sutter movable objective microscope under the control of the ScanImage software package (V. Iyer, Janelia Farms). Scanning was performed at 4 fps using a Chameleon Ultra II laser (Coherent), tuned to 940 nm, and focused through a 16x/0.8NA water immersion lens (Nikon). Emitted light was collected with an HQ535/50 filter (Chroma) and GaAsP photomultiplier tubes (Hamamatsu). Images were acquired at a resolution of 128 × 128 or 256 × 256 pixels. Apical tuft dendrites in layer 1 were imaged at depths of 40-80 μm from the pial surface, and Layer 2/3 and 4 somata were imaged at 200-300 μm and 400-600 μm, respectively.

QUANTIFICATION AND STATISTICAL ANALYSIS

Movies were motion corrected using the SIMA image processing package (Kaifosh et al., 2014). Spatial and temporal components for individual dendrites were extracted using large-scale sparse non-negative matrix factorization (Pnevmatikakis et al., 2016). This method inherently corrects for background signal. Data were analyzed using custom-written routines implemented in MATLAB. Comparisons of frame-averaged calcium signals were performed based upon the average peak amplitude from each session for the time period of 2 s following each behavioral event type. Whisker angle was computed over 150-ms windows and isolated whisking bouts were classified as whisker angle change greater than 2 standard deviations above the mean, with a 1 s lockout. Random reward responsive dendrites were determined based upon proportions of calcium events following random inter-trial interval rewards versus calcium events during inter-trial intervals without random rewards. Dendrites were categorized as reward tracking if the slope of the latency of the second peak in calcium response after the lever lift was significantly related to reward delay time (linear regression). Proportions were compared using a normal approximation to a binomial distribution, and means were compared using t tests.

DATA AND SOFTWARE AVAILABILITY

Data and software are available upon request to the Lead Contact.

Cell Reports, Volume *26*

Supplemental Information

Reinforcement Learning Recruits
Somata and Apical Dendrites
across Layers of Primary Sensory Cortex

Clay O. Lacefield, Eftychios A. Pnevmatikakis, Liam Paninski, and Randy M. Bruno

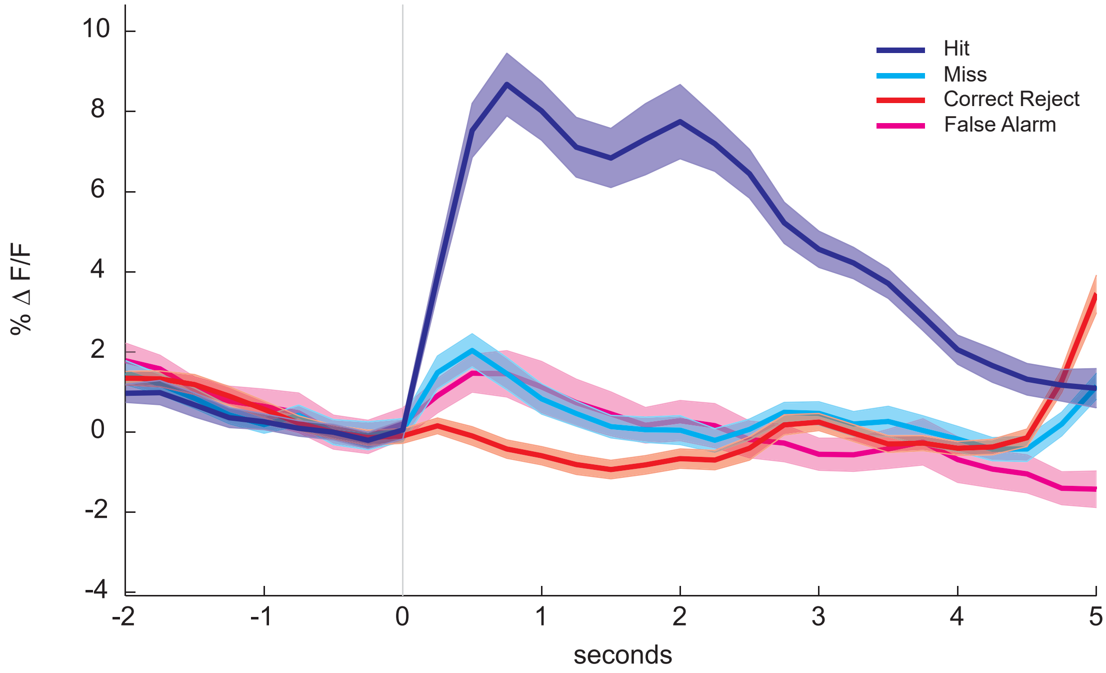

Figure S1, related to Figure 1: Trial averaged whole-frame calcium responses from all four trial outcomes (same data as Fig. 1F).

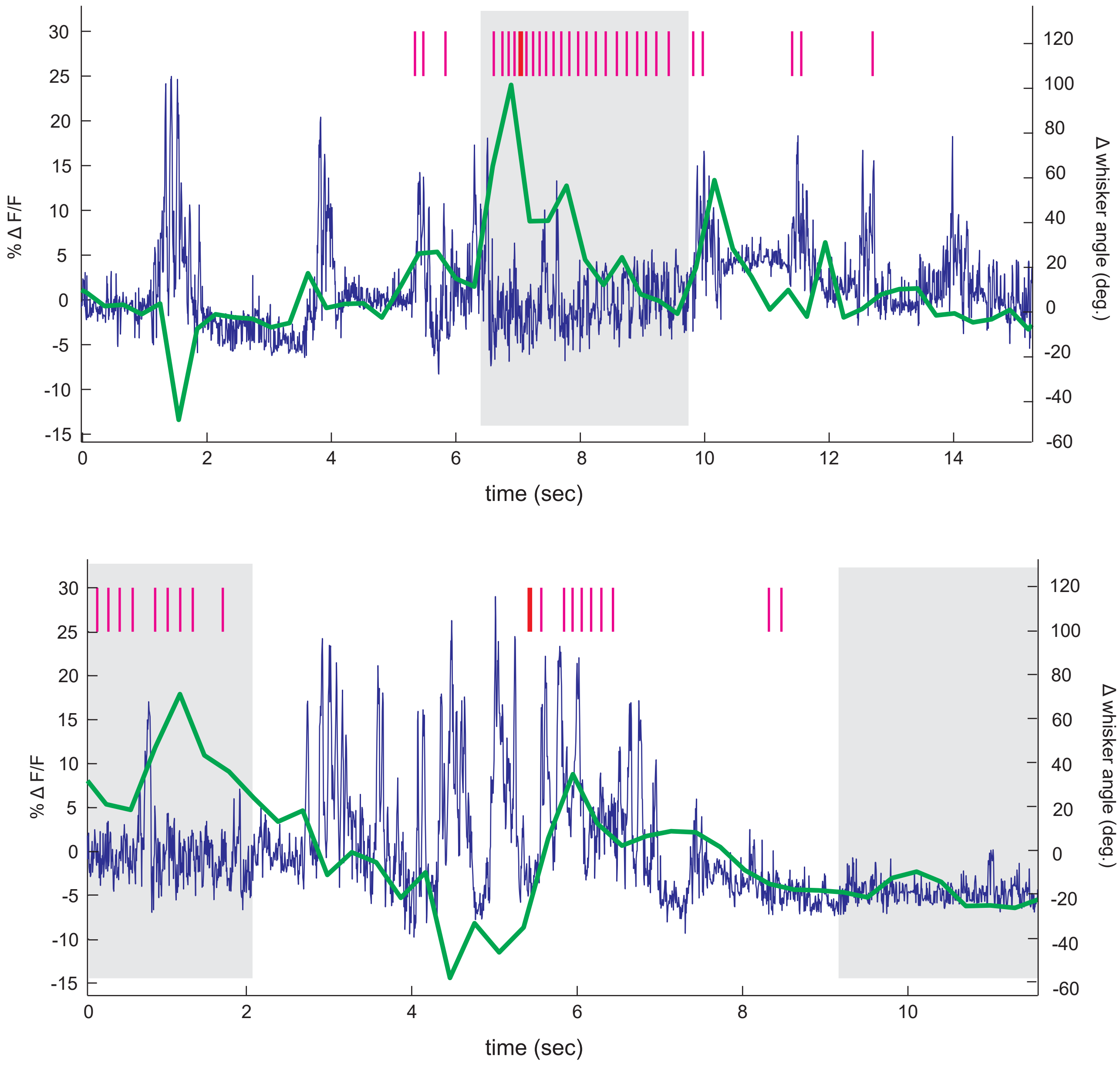

Figure S2, related to Figure 2: Whisker angle (blue) and Layer 5 dendritic calcium (green) during a single rewarded Go trial (top) and random ITI reward (bottom). Gray areas represent trial epochs, while red ticks indicate reward times and magenta ticks indicate lick times. Note that in bottom panel, spontaneous whisking during ITI is accompanied by decreases in calcium levels until the onset of the random ITI reward, which is accompanied by an increase in calcium influx.

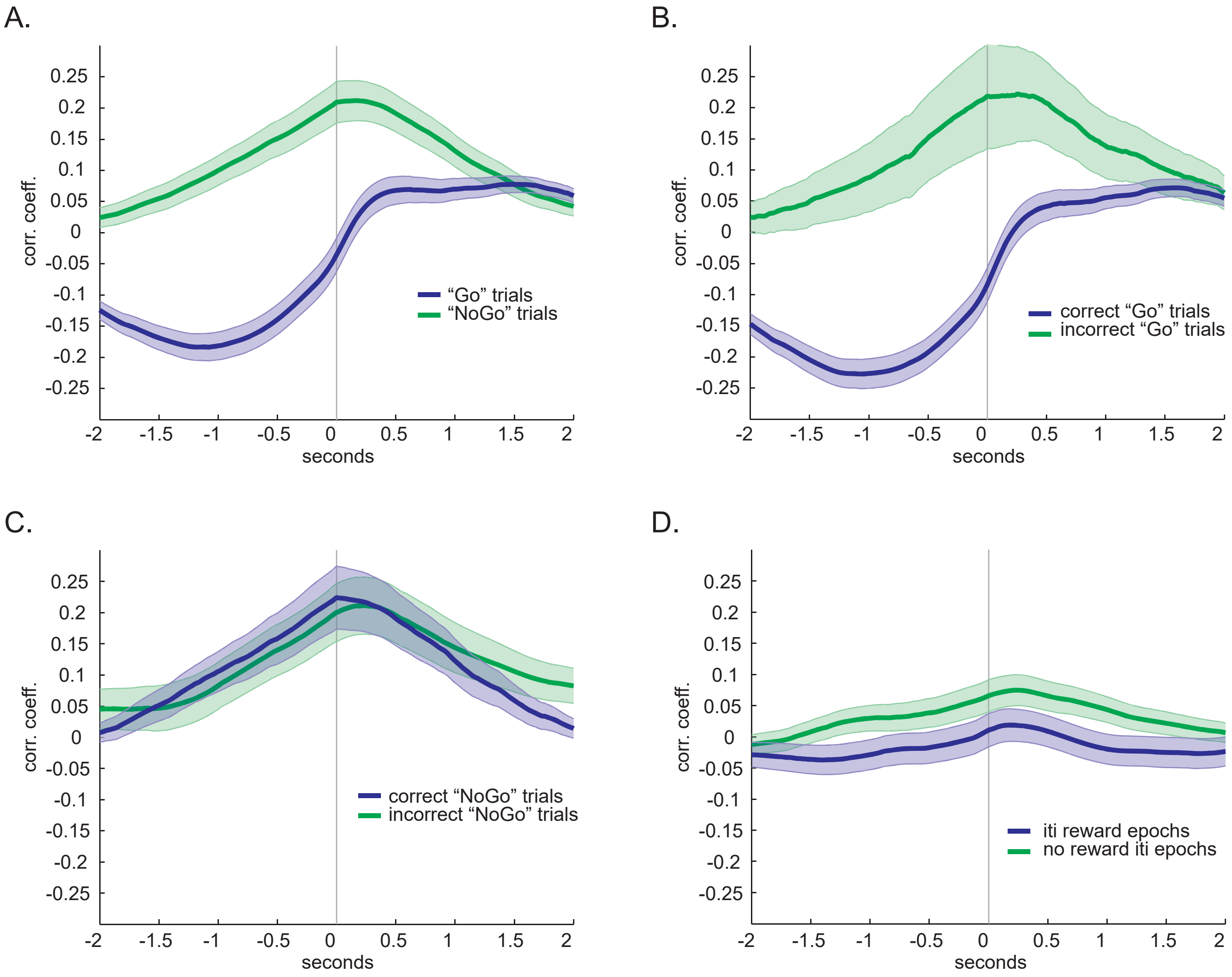

Figure S3, related to Figure 2: Whisking-calcium correlations during behavioral epochs.
(A) Correlation between whisker angle and whole-frame calcium during stimulus "go" trials (blue, n = 204 trials), compared with nogo trials (green, n = 157 trials).
(B) correct/hit (blue, n = 153 trials) vs. incorrect/miss (green, n = 35 trials) "go" trials
(C) correct reject (blue, n = 93 trials) vs. incorrect/false alarm (green, n = 63 trials) "nogo" trials
(D) ITI reward epochs (blue, n = 132 epochs) vs. normal ITI epochs with no random reward (green, n = 229 epochs). In all of these cases, trials with rewards show a decrease in overall correlation between whisking and calcium (n = 6 sessions in 2 mice).

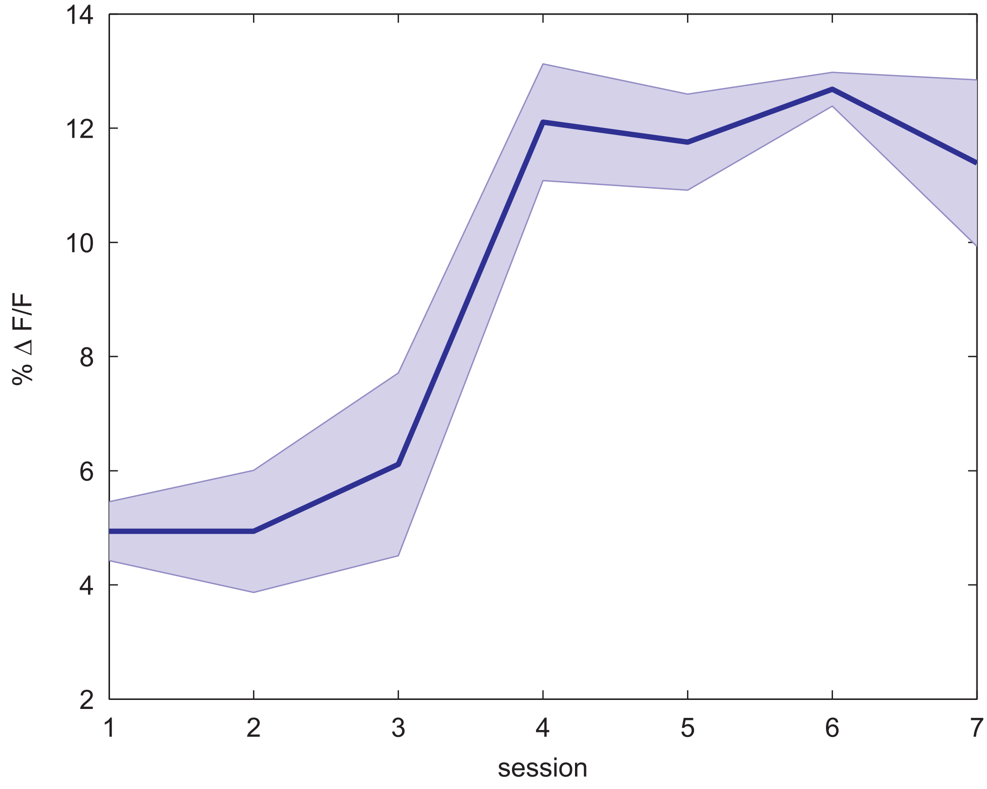

Figure S4, related to Figure 2: Average amplitude of the long-latency peak during trials over sessions (n = 3 animals). Error bound, SEM.

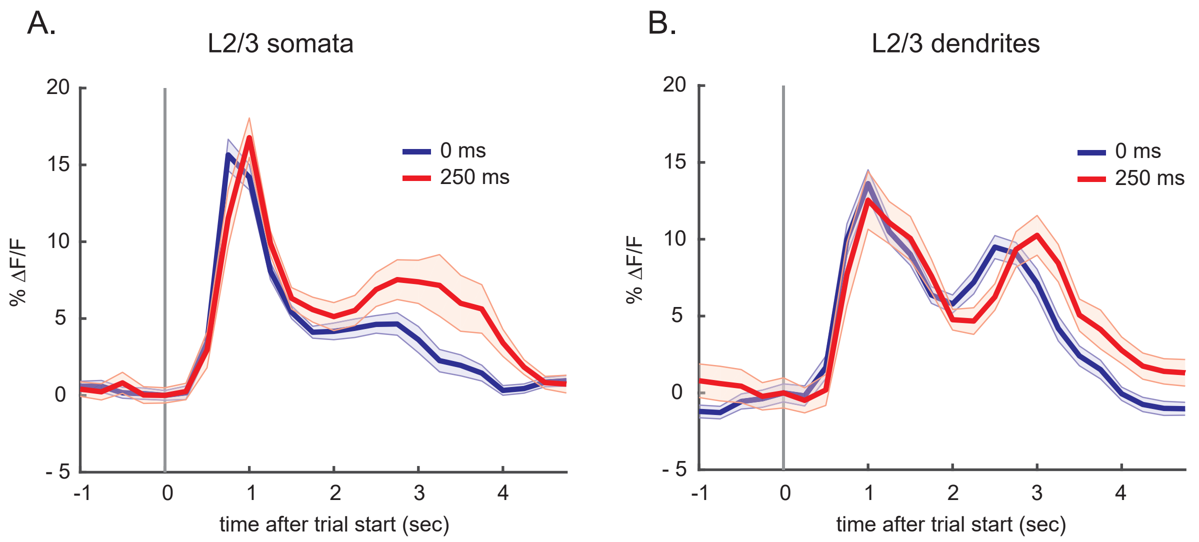

Figure S5, related to Figure 4: Reward tracking in L2/3 somata and dendrites.
(A) Whole-frame calcium signals in L2/3 somata to pole/”Go” trial rewards delayed by 0ms (blue) or 250ms (red).
(B) Whole-frame calcium signals in L2/3 dendrites to pole/”Go” trial rewards delayed by 0ms (blue) or 250ms (red).
